# Overcoming diagnostic challenges in constrictive pericarditis: why volume is important?

**DOI:** 10.1007/s10554-024-03235-3

**Published:** 2024-08-31

**Authors:** Bárbara Lacerda Teixeira, Francisco Albuquerque, Isabel Cardoso, Vera Ferreira, António Fiarresga, Filipe Cardoso, Ana Galrinho, Sílvia Aguiar Rosa, Boban Thomas, Rui Cruz Ferreira

**Affiliations:** 1 Cardiology Department, Hospital de Santa Marta, Centro Hospitalar Universitário de Lisboa Central, Rua de Santa Marta N.º 50, Lisbon, 1169-024 Portugal; 2 Transplant Unit, Hospital Curry Cabral, Centro Hospitalar Universitário de Lisboa Central, Lisbon, Portugal; 3 University Hospital Kerry, Tralee, Republic of Ireland

**Keywords:** Constrictive pericarditis, Chronic liver disease, Cardiac magnetic resonance, Multimodality Imaging, Right heart catheterization

## Abstract

A 65-year-old male with chronic liver disease and refractory ascites was being evaluated for liver transplant, when constrictive pericarditis (CP) was suspected. Initial diagnostics were inconclusive due to overdiuresis. After suspension of diuretics, cardiac magnetic resonance confirmed CP, leading to successful pericardiectomy and normalization of liver function, emphasizing volume status and multimodality imaging role in CP diagnosis.

## Case description

**Fig. 1:**
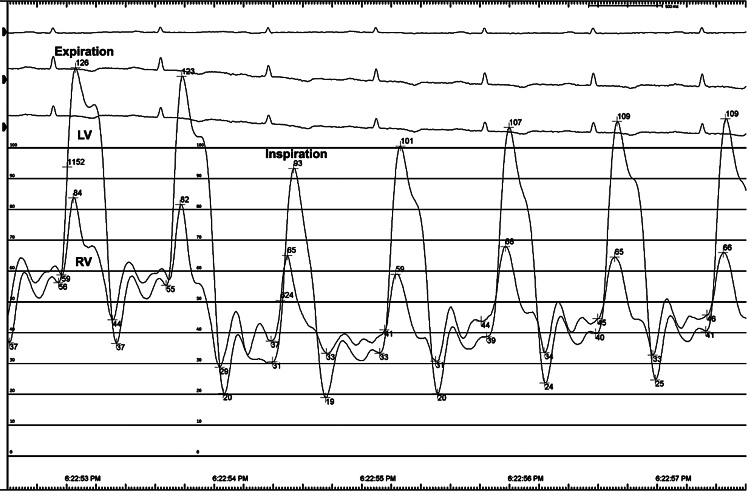
Right heart catheterization (RHC) showing the simultaneous right and left heart ventricular curves. In constrictive pericarditis, exaggerated ventricular interdependence in RHC should demonstrate that the area under the right ventricular systolic pressure curve increases during inspiration, whereas the area under the left ventricular systolic pressure curve decreases. In our patient, although the area under the left ventricular pressure curves decreases slightly, there isn’t a significant change in the right ventricular ones, even after fluid challenge

**Fig. 2:**
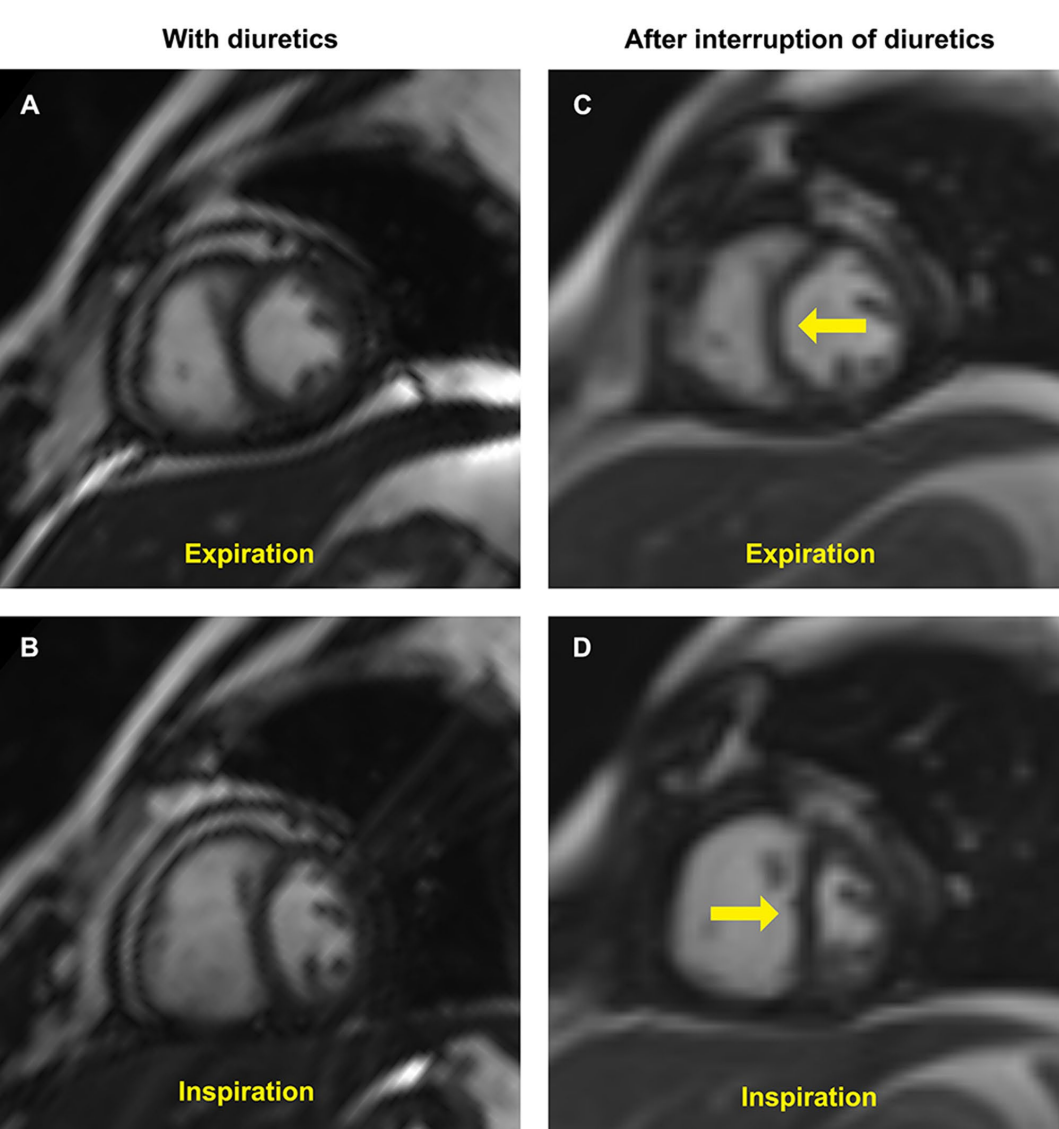
Absence of exaggerated ventricular interdependence on free-breathing protocol of cardiac magnetic resonance (CMR) (**A** and **B**) due to marked diuresis. After the interruption of diuretics for one week, a clear phenomenon of exaggerated ventricular interdependence is seen in CMR images (**C** and **D**), with ventricular septal shift with respiration cycle: rightward shift of septum during expiration (**C**) and leftward shift of septum during inspiration (**d**)

A 65-year-old male with chronic liver disease and cirrhosis under evaluation for liver transplant, with a presumptive diagnosis of cirrhosis secondary to nonalcoholic steatohepatitis, had ascites refractory to increasing doses of diuretics. Liver biopsy, liver *Doppler* ultrasound and magnetic resonance imaging were not consistent with etiology. Transthoracic echocardiogram showed annulus *reversus* and marked respiratory variation of the pulsed wave *Doppler* of mitral and tricuspid inflow velocities suggesting constrictive pericarditis (CP), and the patient was referred to our cardiology outpatient clinic. Further work-up with right heart catheterization (RHC) showed very elevated right atrium pressure (35 mmHg), near equalisation of right and left ventricles end-diastolic pressures (36 and 41 mmHg, respectively), with square-root sign. However, it wasn’t able to show dissociation between pulmonary capillary wedge pressure and left ventricle end-diastolic pressure with respiration nor exaggerated ventricular interdependence (Fig. 1), which couldn’t confirm the diagnosis of CP. An initial cardiac magnetic resonance (CMR) demonstrated thickened pericardium, without pericardial effusion, but was also inconclusive for exaggerated ventricular interdependence. Although the RHC and CMR were carried out after fluid challenge, the patient was taking high doses of diuretics and our cardiology team believed the overdiuresis masked the constrictive physiology. Due to the rapid deterioration of liver function tests and the urgent need to decide on the patient’s eligibility for a liver transplant, it was decided to discontinue diuretics and repeat CMR. After one week of interruption of diuretics, a second CMR demonstrating pericardial thickening, exaggerated ventricular interdependence with ventricular septal shift and diastolic interventricular septal bounce (Fig. 2), gave the answers to our dilemma. The patient underwent pericardiectomy, which resolved his symptoms of fluid overload, and the liver function tests slowly returned to normal. Two years later, the patient remains asymptomatic and off the liver transplant list.

## Discussion

In the setting of a chronic liver disease, with ascites and abnormal liver function tests, the diagnosis of CP can be overlooked. CP is characterized by two pathophysiological abnormalities: exaggerated ventricular interdependence and intrathoracic- intracardiac pressure dissociation.[1] These abnormalities lead to dynamic changes with respiration, which underlie the characteristic signs observed in both invasive and non-invasive diagnostic tests and contribute to the patient’s symptoms.[2] These classic signs of constriction are more pronounced in euvolemic patients.[3] If these signs are absent in individuals suspected of CP, and volume depletion is present, it is recommended to reassess after fluid replacement or, as in our case, diuretic adjustment. Although RHC remains the gold standard for confirmation of constrictive physiology, multimodality imaging, including CMR, may be useful in uncertain diagnosis. [1]

## Conclusion

The diagnosis of CP can be challenging and requires a high level of suspicion. Patients with CP often receive high doses of diuretics for their symptoms, which can obscure the typical signs of the condition in both RHC and imaging studies. Clinicians often need to optimize fluid status in these patients before diagnostic testing to enhance diagnostic accuracy. Additionally, CMR may be essential for a definitive diagnosis.

## Data Availability

No datasets were generated or analysed during the current study.
